# Diet and diet combined with chronic aerobic exercise decreases body fat mass and alters plasma and adipose tissue inflammatory markers in obese women

**DOI:** 10.1186/1546-0096-13-S1-P194

**Published:** 2015-09-28

**Authors:** N Lakhdar, M Denguezli, M Zaouali, A Zbidi, Z Tabka, A Bouassida

**Affiliations:** 1 Research Unit of Sportive Performance and Physical Rehabilitation, High Institute of Sports and Physical Education, Physiology, El Kef, Tunisia; 2 High Institute of Sports and Physical Education, Research Unit of Sportive Performance and Physical Rehabilitation, El Kef, University of Jendouba, Tunisia; 3 Laboratory of Cardio-Circulatory, Respiratory, Metabolic and Hormonal Adaptations to Muscular Exercise, Faculty of Medicine Ibn El Jazzar, Physiology, Sousse, Tunisia; 4 Laboratory of Cardio-Circulatory, Respiratory, Metabolic and Hormonal Adaptations to Muscular Exercise, Physiology, Sousse, Tunisia; 5 Research Unit of Sportive Performance and Physical Rehabilitation, Physiology, El Kef, University of Jendouba, Tunisia, Tunisia

## 

The purpose of this study was to investigate the effect of 6 months aerobic exercise and diet alone or in combination on markers of inflammation (MOI) in circulation and in adipose abdominal tissue (AT) in obese women. Thirty obese subjects were randomized into a 24 weeks intervention: 1) exercise (EX), 2) diet (DI) and *3*) exercise and diet (EXD). Blood samples were collected at baseline, after 12 wk and 24 wk. AT biopsies were obtained only at baseline and after 24 wk. In the EXD and DI groups the fat loss was after 12 wk -13.74% and -7.8% (P<0.01) and after 24 wk -21.82% and -17% (P<0.01) with no changes in the EX group. After 12 and 24 wk, VO_2_ max was increased by 21.81-39.54% (P<0.05) in the EXD group and 18.09-40.95% in the EX group with no changes in the DI group. In the EXD and DI groups, circulating levels of TNF-a and IL-6 were decreased after 24 wk for both groups (P<0.01). No changes in the EX group. HOMA-R decreased (P<0.05) only after 24 wk in the EXD group. In AT biopsies, subjects in the EXD and DI groups exhibited a significant decrease in MO (P<0.01 for all). No changes in AT biopsies were found in the EX group. In conclusion, chronic aerobic exercise was found to have no effects on circulating and AT MOI despite an increased VO_2_ max. Rather important body composition modifications were found to have beneficial effects on circulating and AT MOI in these obese women.

